# Phylogenomic Based Comparative Studies on Indian and American Commensal *Staphylococcus epidermidis* Isolates

**DOI:** 10.3389/fmicb.2018.00333

**Published:** 2018-02-27

**Authors:** Shikha Sharma, Vasvi Chaudhry, Sanjeet Kumar, Prabhu B. Patil

**Affiliations:** Bacterial Genomics and Evolution Laboratory, CSIR-Institute of Microbial Technology, Chandigarh, India

**Keywords:** *Staphylococcus epidermidis*, commensal, geographic, markers, pathogen, phylogeographic, evolution, phylogenomics

## Abstract

*Staphylococcus epidermidis* is a prominent commensal member of human skin microbiome and an emerging nosocomial pathogen, making it a good model organism to provide genomic insights, correlating its transition between commensalism and pathogenicity. While there are numerous studies to understand differences in commensal and pathogenic isolates, systematic efforts to understand variation and evolutionary pattern in multiple strains isolated from healthy individuals are lacking. In the present study, using whole genome sequencing and analysis, we report presence of diverse lineages of *S. epidermidis* isolates in healthy individuals from two geographically diverse locations of India and North America. Further, there is distinct pattern in the distribution of candidate gene(s) for pathogenicity and commensalism. The pattern is not only reflected in lineages but is also based on geographic origin of the isolates. This is evident by the fact that North American isolates under this study are more genomically dynamic and harbor pathogenicity markers in higher frequency. On the other hand, isolates of Indian origin are less genomically dynamic, harbor less pathogenicity marker genes and possess two unique antimicrobial peptide gene clusters. This study provides a basis to understand the nature of selection pressure in a key human skin commensal bacterium with implications in its management as an opportunistic pathogen.

## Introduction

*Staphylococcus epidermidis* is coagulase-negative staphylococcus (CONS) commensal member of human skin microbiota inhabiting skin since millions of years (Rogers et al., 2009). As a key member of skin microbiome, its role in skin homeostasis is widely assumed (Kong et al., 2012; Skabytska and Biedermann, 2016). There are many studies showing its role in immune system modulation (Lai et al., 2009, 2010; Belkaid and Tamoutounour, 2016; Egert et al., 2017). It is also considered crucial for skin microbiota because of its ability to inhibit the growth of notorious skin pathogen, *Staphylococcus aureus* (Otto et al., 1999; Heikkilä and Saris, 2003; Iwase et al., 2010). It produces antimicrobial peptides, which inhibit the growth of pathogenic strains (Nakatsuji et al., 2017). However, in recent decades, *S. epidermidis* has emerged as an opportunistic pathogen, as a frequent cause of nosocomial infections (Hidron et al., 2008; Otto, 2009) in immunocompromised patients or patients with indwelling medical devices (Marr et al., 1997; Varani et al., 2009; Percival et al., 2015). Abundance of *S. epidermidis* on skin enhances the probability of contamination of indwelling devices through formation of biofilms. Presence of biofilms on these medical devices interrupts the proper functioning of the devices and cause bacteremia (Costerton et al., 1999; Mishra et al., 2015; Otto, 2017). *Staphylococcus epidermidis* is the major causative agent for blood stream infections, vascular graft, central nervous system shunt, prosthetic joint infections, prosthetic valve endocarditis, and other biomedical device-related infections (Widerström et al., 2012a). It has also been reported to play a role in neonatal sepsis (Sgro et al., 2011). Management of *S. epidermidis* is further challenged by the presence of antibiotic resistance genes (Archer, 1978; Raad et al., 1998; Gill et al., 2005; Dave et al., 2011; Widerström et al., 2012b), thereby contributing to multi-drug resistance forms of the species (Rabaud and Mauuary, 2001; Miragaia et al., 2005). These strains may also transfer them horizontally to its pathogenic relative, *S. aureus* (Méric et al., 2015). Thus, management of this commensal yet an opportunistic pathogen is crucial in both community and clinical settings.

One of the major challenges in treating pathogenic *S. epidermidis* is the lack of proper demarcation or markers to differentiate among commensal and pathogenic isolates (Galdbart et al., 2000; Grice and Segre, 2011). Some studies based on MLST analysis and other genetic studies highlights genes among *S. epidermidis* isolates of hospital environment and from those obtained outside of it (Galdbart et al., 2000). Some genes, including, IS*256* (insertion sequence) (Kozitskaya et al., 2004), *mecA* (methicillin resistance gene) (Lazaris et al., 2017; Qin et al., 2017; Takizawa et al., 2017) and *icaA* (biofilm biosynthesis cluster *ica* gene) (Grice and Segre, 2011) are found to be more frequently present in pathogenic strains and are also shown to contribute to their pathogenicity. These genes are also present in pathogenic strain, RP62A and hence, are proposed by some studies as potential markers to delineate pathogenic strains of *S. epidermidis* (Galdbart et al., 2000; Gu et al., 2005). Some other genes viz. *fdh* and *arsD*, encoding for “formate dehydrogenase” “arsenic resistance gene” respectively, are observed to be frequent in commensal strains (Conlan et al., 2012). Higher frequency of these genes in their *S. epidermidis* isolates based on their origin hints in their ability to resolve the demarcation of commensal and pathogenic *S. epidermidis*. However, their full scope as markers is yet to be exploited.

There are numerous studies highlighting the clinical importance of this bacterium. However, no studies have been carried out so far emphasizing its phylogenomic aspect that can help in understanding the diversity among multiple isolates of a healthy individual. While earlier studies focused on the differential features among commensal and pathogenic isolates (Harris et al., 2016; Post et al., 2017), this study mainly pinpoints diversity and evolution among commensal isolates of diverse geographic origin.

Since *S. epidermidis* inhabiting the skin through ages, there might be ongoing selection on isolates within and between healthy individuals. In a recent publication, we reported that *S. epidermidis* isolates from diverse habitats like plants, rodents and human shown remarkable variation suggesting the ongoing selection in this species (Chaudhry and Patil, 2016). Moreover, there are studies on inter-strain variation, based on pangenome analysis of *S. epidermidis* isolates from different body sites (Conlan et al., 2012). However, there are no studies on inter-strain variation of *S. epidermidis* isolates living on healthy individuals from diverse geographic origins, which might be due to ongoing selection on isolates within and between healthy individuals. Advent of next generation sequencing and large-scale genome based studies are allowing us to capture inter-strain variation at unprecedented details.

In the present study, we report whole genome sequencing of twenty-eight *S. epidermidis* isolates from different body parts of skin of healthy individuals from India, analyzed with twenty-one *S. epidermidis* isolates from different body parts of skin of healthy individuals from North America (Conlan et al., 2012).

## Materials and methods

### Ethics, consent, and permissions

The study was approved by the Institutional Ethics Committee (Human), CSIR-Institute of Microbial Technology, Chandigarh (India) [Project no. 12IEC/1/9-2014] and informed consent was obtained from the individuals.

### Bacterial strains

Twenty-eight *S. epidermidis* isolates (SEI), from different body sites of healthy individuals, viz., retro-auricular crease, nares, antecubital fossa and opisthenar area of hand, from India were isolated from four healthy individuals. Isolations were done as described by Farran et al. (2013). Pre-moistened saline (0.85% NaCl) swabs were rubbed with skin of the healthy individuals and transferred to TSB (Tryptic Soy Broth) for enrichment. Inoculated media was than incubated at 37°C in a shaker ~200 rpm and growth was observed after 16-18 hrs. Overnight grown culture was then streaked onto MSA (mannitol salt agar) plates and was incubated at 37°C for ~36–48 h. Pink and white color colonies were observed on agar plates indicating *S. epidermidis* and *S. aureus* populations respectively. Pure cultures of *S. epidermidis* were obtained and identified by 16S rRNA sequencing using amplification primers 27F (AGAGTTTGATCMTGGCTCAG) and 1492R (GGTTACCTTGTTACGACTT), and further 16S rRNA gene sequence was used for characterization using EZ-taxon server (http://www.ezbiocloud.net/eztaxon; Kim et al., 2012). For preservation, 15% glycerol stocks of the pure culture of each isolate was prepared and maintained at −80°C. Genome sequences of type strain, MTCC3382 (accession number: NZ_LILE01000007) and reference strains, RP62A (accession number: NC_002976), ATCC12228 (accession number: NC_004461) were taken from NCBI for detailed analysis.

Twenty-one isolates of western origin (SEA) used in this study were taken from a similar study where multiple *S. epidermidis* isolates were extracted from different body sites of several healthy individuals (Table 1; Conlan et al., 2012).

**Table 1:** Details of the *S. epidermidis* isolates extracted from different body sites of healthy individuals, of two different geographical locations.

	**Isolate**	**Site and source**	**Location**	**Size (Mb)**	**GC (%)**	**CDS**	**rRNA**	**tRNA**	**Accession number**	**References**
1	VSE57	Retroauricular crease, F1	Chandigarh, India	2.4	32	2258	9	58	MLXL	this study
2	VSE46	Retroauricular crease, F1	Chandigarh, India	2.4	32	2272	5	57	MLXE	this study
3	VSE43	Antecubital Fossa, F1	Chandigarh, India	2.4	31.9	2581	5	57	MLXD	this study
4	VSE52	Nares, F1	Chandigarh, India	2.4	32	2256	8	57	MLXI	this study
5	VSE50	Antecubital Fossa, F1	Chandigarh, India	2.39	32	2241	10	58	MNBF	this study
6	VSE44	Retroauricular crease, F1	Chandigarh, India	2.4	32	2331	9	58	MNAB	this study
7	SE55	Nares, F1	Chandigarh, India	2.4	32	2304	8	56	LUBV	this study
8	VSE35	Retroauricular crease, F2	Chandigarh, India	2.5	32.3	2330	9	58	MLXN	this study
9	VSE37	Retroauricular crease, F2	Chandigarh, India	2.4	31.9	2268	7	56	MLWZ	this study
10	VSE42	Retroauricular crease, F2	Chandigarh, India	2.4	32	2253	7	47	MLXC	this study
11	VSE53	Antecubital Fossa, F2	Chandigarh, India	2.4	32	2282	7	44	MLXJ	this study
12	VSE58	Retroauricular crease, F2	Chandigarh, India	2.4	31.9	2271	12	33	MLXM	this study
13	VSE48	Nares, F2	Chandigarh, India	2.3	32	2178	7	34	MLXG	this study
14	VSE54	Antecubital Fossa, F2	Chandigarh, India	2.4	31.9	2255	11	57	MLXK	this study
15	VSE39	Nares, F2	Chandigarh, India	2.4	31.9	2264	10	58	MLXA	this study
16	SE40	Nares, F2	Chandigarh, India	2.48	31.9	2279	6	50	LUBM	this study
17	VSE41	Nares, F2	Chandigarh, India	2.4	32	2256	8	58	MLXB	this study
18	VSE49	Nares, M1	Chandigarh, India	2.4	32	2256	8	58	MLXH	this study
19	SE51	Retroauricular crease, M1	Chandigarh, India	2.47	31.9	2275	6	53	LUBR	this study
20	SE45	Retroauricular crease, M1	Chandigarh, India	2.48	31.9	2272	8	50	LUBH	this study
21	VSE56	Antecubital Fossa, M1	Chandigarh, India	2.4	31.9	2302	11	31	LUBG	this study
22	VSE36	Nares, M1	Chandigarh, India	2.45	32.5	2411	18	61	MSYS	this study
23	VSE47	Retroauricular crease, M1	Chandigarh, India	2.4	32	2249	6	58	MLXF	this study
24	VSE1	Opisthenar hand, F3	Chandigarh, India	2.54	31.9	2315	5	54	LGVM	this study
25	VSE2	Opisthenar hand, F3	Chandigarh, India	2.42	32.1	2242	4	55	LGVI	this study
26	VSE3	Opisthenar hand, F3	Chandigarh, India	2.42	32.1	2258	4	56	LGVJ	this study
27	VSE4	Opisthenar hand, F3	Chandigarh, India	2.42	32.1	2263	4	58	LGVK	this study
28	VSE5	Opisthenar hand, F3	Chandigarh, India	2.48	32	2300	5	52	LGVL	this study
29	NIHLM001	Alar crease, HV1	Bethesda, MD, US	2.6	31.84	2535	21	66	AKHC	Conlan et al., 2012
30	NIHLM003	Umbilicus, HV1	Bethesda, MD, US	2.6	31.96	2481	23	59	AKHB	Conlan et al., 2012
31	NIHLM008	Alar crease, HV3	Bethesda, MD, US	2.4	32	2300	21	65	AKHA	Conlan et al., 2012
32	NIHLM015	Manubrium, HV3	Bethesda, MD, US	2.4	32.12	2313	27	64	AKGZ	Conlan et al., 2012
33	NIHLM018	Umbilicus, HV3	Bethesda, MD, US	2.5	31.99	2339	24	60	AKGY	Conlan et al., 2012
34	NIHLM031	plantar heel, HV4	Bethesda, MD, US	2.5	31.94	2455	23	59	AKGX	Conlan et al., 2012
35	NIHLM020	axilla, HV4	Bethesda, MD, US	2.4	32.6	2263	19	67	AKGW	Conlan et al., 2012
36	NIHLM021	axilla, HV6	Bethesda, MD, US	2.5	32.1	2408	18	66	AKGV	Conlan et al., 2012
37	NIHLM023	toe web, HV6	Bethesda, MD, US	2.5	31.96	2430	18	70	AKGU	Conlan et al., 2012
38	NIHLM037	glabella, HV9	Bethesda, MD, US	2.5	32.06	2333	19	64	AKGT	Conlan et al., 2012
39	NIHLM039	retroauricular crease, HV9	Bethesda, MD, US	2.5	31.92	2404	16	59	AKGS	Conlan et al., 2012
40	NIHLM040	retroauricular crease, HV9	Bethesda, MD, US	2.6	31.93	2458	22	58	AKGR	Conlan et al., 2012
41	NIHLM049	gluteal crease, HV10	Bethesda, MD, US	2.5	31.89	2355	21	62	AKGQ	Conlan et al., 2012
42	NIHLM053	hypothenar palm, HV10	Bethesda, MD, US	2.5	32	2479	18	60	AKGP	Conlan et al., 2012
43	NIHLM057	occiput, HV10	Bethesda, MD, US	2.5	32.04	2459	19	66	AKGO	Conlan et al., 2012
44	NIHLM061	nare, HV10	Bethesda, MD, US	2.6	32.02	2444	19	62	AKGN	Conlan et al., 2012
45	NIHLM067	axilla, HV2	Bethesda, MD, US	2.4	32.14	2269	22	62	AKGM	Conlan et al., 2012
46	NIHLM070	alar crease, HV2	Bethesda, MD, US	2.5	31.98	2436	21	59	AKGL,	Conlan et al., 2012
47	NIHLM087	Nare, HV6	Bethesda, MD, US	2.5	32.1	2360	24	60	AKGK	Conlan et al., 2012
48	NIHLM088	Nare, HV6	Bethesda, MD, US	2.5	31.85	2434	21	67	AKGJ	Conlan et al., 2012
49	NIHLM095	gluteal crease, HV6	Bethesda, MD, US	2.5	32.07	2400	20	59	AKGI0	Conlan et al., 2012

### Genome sequencing, assembly and annotation

Cells were revived from −80°C stocks and DNA was isolated using ZR Fungal/Bacterial DNA isolation kit (Zymo Research Corporation, Orange, CA, USA) as per the instruction manual. The purity, quality and quantity of purified genomic DNA were assessed using Nanodrop (Thermo Scientific, MA, USA), agarose gel electrophoresis and Qubit 2.0 Fluorometer (Invitrogen, Carlsbad, CA, USA) respectively. Illumina DNA sequencing libraries was prepared by using Nextera XT sample preparation kit (Illumina, Inc., San Diego, CA, USA) with dual indexing adapters, as per the instruction manual and prepared library was sequenced on in-house Illumina MiSeq platform (Illumina, Inc., San Diego, CA, USA) using paired end sequencing kits. Adapter trimming was carried out automatically by MiSeq Control Software (MCS) and additional adapter contamination identified by NCBI server was removed by manual trimming. De novo assembly of the sequences was carried out using CLC genomic workbench v7.5 (CLC Bio-Qiagen, Aarhus, Denmark). 16S rRNA sequences were extracted from the assembled genomes using RNAmmer 1.2 server (Lagesen et al., 2007) and were characterized by Ez-taxon server. tRNA was calculated by tRNAScan-SE (Lowe and Eddy, 1997). Sequences were annotated using NCBI Prokaryotic Genome Annotation Pipeline (http://www.ncbi.nlm.nih.gov/genome/annotation_prok/) and Rapid Annotation System Technology (RAST) pipeline (Aziz et al., 2008). Genome sequences of all 28 isolates sequenced under this study are submitted in NCBI GenBank and are summarized into Table 1.

### Genome based taxonomy and phylogenomics

Phylogenetic analysis of the isolates was carried out by constructing a tree based on 400 universal protein by PhyloPhlAn version 0.99 (Segata et al., 2013) and was represented by interactive tree of life (iTOL) version 3 (Letunic and Bork, 2006). For genome similarity assessment, BLAST-based average nucleotide identity (ANIb) values were calculated by using JSpecies (Richter and Rosselló-Móra, 2009).

### Multilocus sequence typing (MLST)

Sequence type (ST) and consecutive allelic profile of all genomes was elucidated from an online platform by Center for genomic epidemiology (Larsen et al., 2012; http://cge.cbs.dtu.dk/services/MLST). This tool utilizes the prescribed MLST scheme of seven genes. Two genes, *gtr* and *tpi*, were not detected in genomes, VSE3, VSE43 and VSE48, VSE50, respectively. Reads for these genomes were re-assembled in Spades version 3.11 (Bankevich et al., 2012), required genes were extracted and ST was analyzed. Generated STs were confirmed in MLST database (Aanensen and Spratt, 2005; http://www.mlst.net). Novel allele profile was submitted to PubMLST database (Jolley et al., 2004; http://pubmlst.org/sepidermidis/) to obtain the unique and novel ST information.

### Comparative genome analysis

Comparative subsystem analysis of all isolates was carried out by comparative examination of their RAST annotations. Atypical genomic regions were extracted by using whole genome based Blast based Ring Image Generator (BRIG) tool version 0.95 (Alikhan et al., 2011). All genomes were tried as reference in BRIG analysis, to extract out most significant atypical regions. Observed atypical genomic regions were taken out by Artemis comparisons (Carver et al., 2012). Pangenome analysis was carried for all 49 genomes, out by BPGA v1.3 (Chaudhari et al., 2016). A minimum identity of 50% was used as the cut-off. Number of combinations of genomes considered for analysis were chosen to be 50, which signifies the maximum number of possible clusters for a USEARCH run. USERACH is an integral dependency of BPGA used for clustering. Flower plot diagram used to represent the core and unique gene was constructed using a python script of Matplotlib (Hunter, 2007).

### Genome mining

*In silico* search of candidate virulence genes was carried out in RAST annotated genomes (Aziz et al., 2008) and was further confirmed by BLASTN (Johnson et al., 2008) to analyze genes for commensalism and pathogenicity.

Genomes were mined in antiSMASH tool version 2.0 (Medema et al., 2011) and BAGEL version 3 (van Heel et al., 2013) web tool for examining gene clusters for secondary metabolites, bacteriocin, or lantibiotics. Individual genes of clusters were mapped manually using BLASTN. Prophages were checked through web server PHAST (Phage search tool) (Zhou et al., 2011).

## Results

### Genome sequencing and annotation

Twenty-eight *S. epidermidis* (SE) isolates from different body sites of healthy individuals of Indian origin (SEI) were sequenced (Table S1) and high quality data was obtained with coverage >50x and number of contigs in range, 52–176. Annotation of all isolates revealed CDS ranging from 2,178 to 2,535. Sequencing and annotation details of SEI isolates and 21 *S. epidermidis* isolates of American origin (SEA) from another study (Conlan et al., 2012) are provided in Table 1.

### Phylogenomic and phylogeographic analysis of SEI and SEA isolates

**Figure 1:**
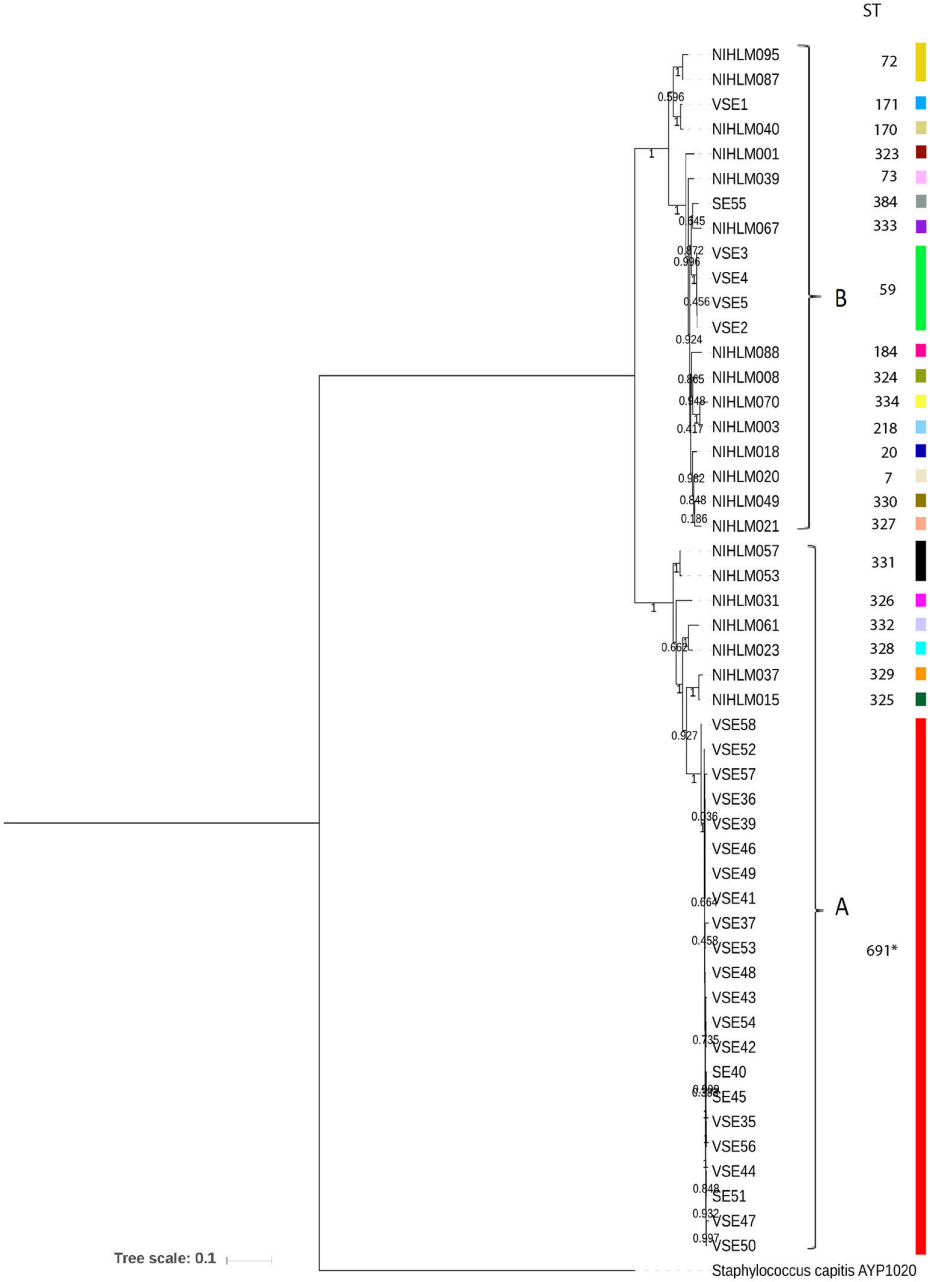
Phylogenetic tree among 49 *Staphylococcus epidermidis* isolates, based on protein sequence of 400 universal housekeeping genes, using MEGA7.10.18. *S. capitis* AYP1020 was used as outgroup of the tree and bootstrap values are highlighted on the branches. STs of all isolates are mentioned against them and isolates with similar STs or clones are represented with same color-coding.

Phylogenetic relationship among 49 SE isolates (Table S2) was elucidated by constructing a phylogenetic tree based on 400 conserved protein sequences (Figure 1). The tree branched into two major clades, “A” and “B.” Majority of SEI isolates were present in “A” clade while SEA isolates were divided uniformly among the two clades.

**Figure 2:**
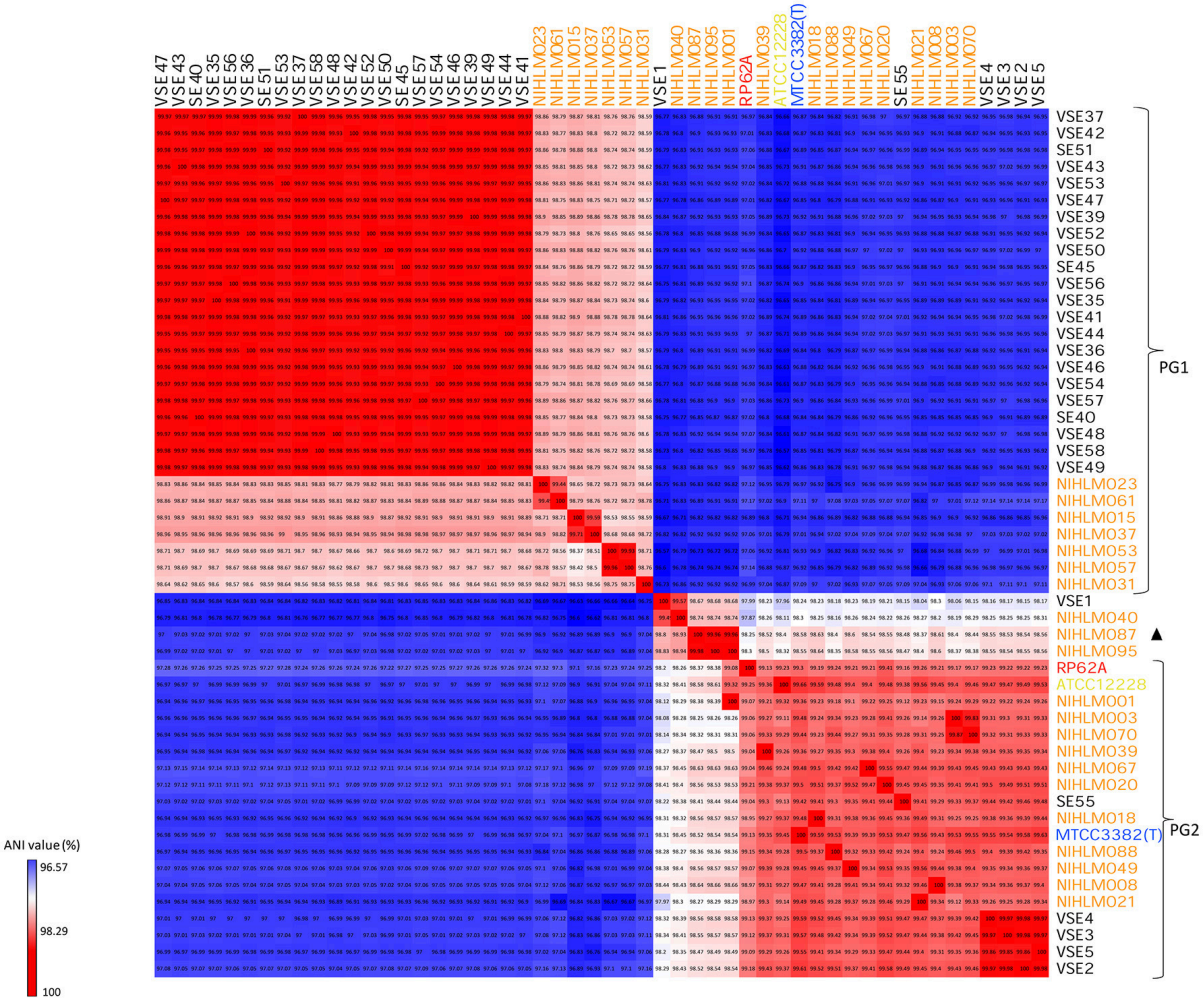
ANI heatmap of SEI and SEA isolates. PG1 and PG2 demonstrates two kind of lineages, phylogenetic group 1 and phylogenetic group 2; Blank triangle represents transitional phase. Orange color text isolates are SEA isolates. Type strain, MTCC 3382 (T) [In blue], pathogenic strain RP62A [In red] and commensal strain, ATCC12228 [In yellow].

Further, genetic relatedness among all 49 isolates was deduced by whole genome based, ANI heatmap tree using type strain, MTCC 3382 (T). Biofilm forming strain, RP62A and non-biofilm forming strain, ATCC12228 were also used as reference strains. All 49 isolates demarcated themselves in two major phylogenetic lineages, PG1 and PG2, and a minor group of four isolates, independent of the habitat, individual or body site from which they were isolated (Figure 2). PG1 lineage of ANI tree corroborated with clade “A” of phylogenetic tree, consisting of the same isolates. PG1 lineage mainly consists of SEI isolates, having ANI > 99% among themselves, forming a single clonal group, while some SEA isolates of the same lineages were comparatively more diverse. PG2 lineage consists of five SEI isolates along with type strain, MTCC 3382 (T), reference strains, ATCC12228, RP62A and other SEA isolates. The minor group represented by four isolates, appeared as a transitional lineage, having intermediate ANI values of PG1 and PG2 lineages.

### Multilocus sequence type (ST) analysis of SEI and SEA genomes

Nineteen Sequence Types (ST) of 21 SEA genomes is already mentioned in the earlier study (Conlan et al., 2012) and ST of SEI isolates were determined in this study (Figure 1). Four STs were observed in SEI isolates. Isolates VSE2-VSE5 belong to ST59, isolate SE55 belong to ST384, and isolate VSE1 belong to ST171. Interestingly all the SEI isolates of PG1 lineage or clade “A,” i.e., VSE35-VSE58 belong to a novel ST691 (Table S3). All the STs are lineage or clade specific and large majority (16 out of 23 STs) belong to clade “B” or PG2 lineage. MLST typing revealed that PG1 lineage isolates are less diverse than PG2 lineage corroborating finding from core genome tree and phylogenomic analysis.

### Phylogenomic and phylogeographic variation in pathogenicity and commensal candidate genes(s) in *S. epidermidis* isolates

**Figure 3:**
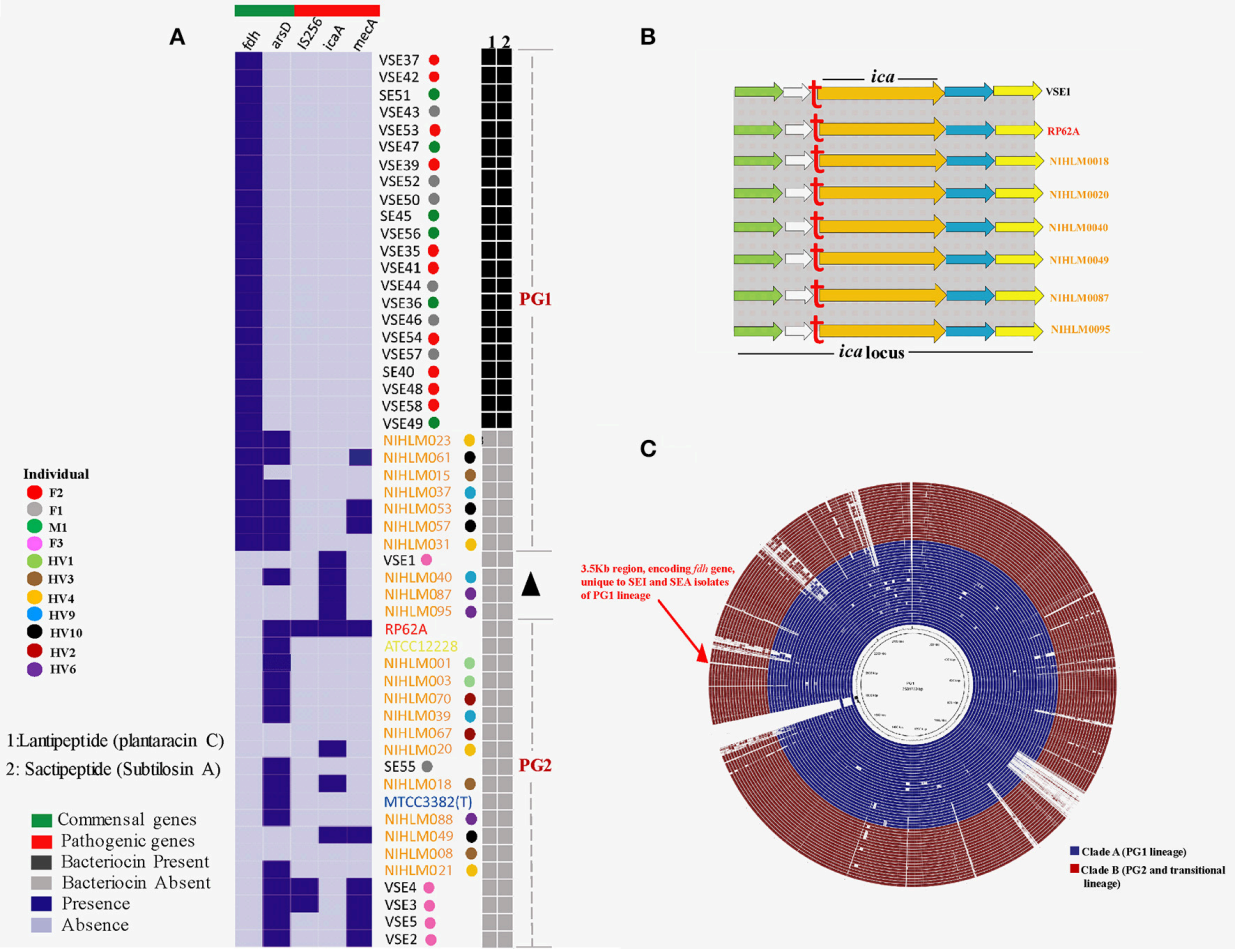
**(A)** *In-silico* mining of potential commensal and pathogenic markers in isolates and type strain. Blue color indicates presence of the corresponding gene, which is estimated as 100% coverage of the gene with identity value >80% and Light blue color indicates absence of the gene, which is estimated by no identity, *e*-value >0 or coverage <5. Different color-coding is representation for different individuals. Details of the individuals are provided in metadata in Table S2. Bacteriocins, lantipeptide and sactipeptide are denoted by 1 and 2 respectively. Their presence or absence is marked by Dark gray and Light gray respectively. **(B)** Complete *ica* cluster along with upstream downstream regions in *icaA* positive isolates. A red “t” indicates presence of tRNA in all isolates. The whole cluster along with downstream, upstream genes is exactly similar in all isolates. Green: MFS transporter, Blank: Hypothetical gene, Blue: Lipase, Yellow: N-acetyltransferase. **(C)** Blast based comparative analysis of 49 *S. epidermidis* isolates by BRIG, taking VSE35 as reference, blue color represents PGI isolates and red color represents PG2 and transitional lineages isolates, irrespective of geographic origin.

Commensal strains marker gene, “*fdh*” was seen to be present in all isolates (SEI and SEA) of lineage PG1. While another gene, *arsD*, was absent from all SEI isolates of PG1 and was present in SEA isolates of the same lineage. Distribution of this gene in PG2 and transitional lineage is uneven and inconsistent with both SEA as well as SEI isolates (Figure 3A).

IS*256*, an insertion sequence, is a marker for pathogenicity (Kozitskaya et al., 2004) in *S. epidermidis* was absent from all isolates of PG1 and transitional lineage and was present only in two SEI isolates of lineage PG2. While *mecA* gene (methicillin resistance) was absent from SEI isolates of lineage PG1 and present in 4 SEI isolates of PG2. On the other hand, three SEA isolates of lineage PG1 and one from PG2 were observed having *mecA* resistance gene.

Biofilm forming, *ica* gene cluster consists of 5 genes, *icaR, icaA, icaD, icaB*, and *icaC*. Most important gene of this cluster is *ica*A, which is considered crucial for biofilm formation and was searched in all genomes. The gene was present in all isolates of transitional lineage and only in 2 isolates of PG2 lineage. Whole cluster of the *ica* was *in silico* mined in all seven genomes along with their loci, which were found to be in complete synteny to each other (Figure 3B).

### Comparative genomics of SEA and SEI isolates

#### Pangenome evolution and diversification

**Figure 4:**
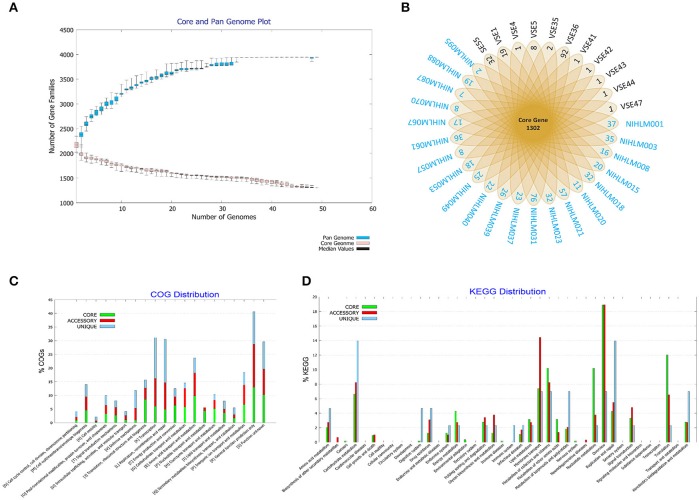
Pangenome profile analysis of 49 *S. epidermidis* isolates. Indian origin isolates are highlighted in black, while American origin isolates are marked in blue **(A)** Pan-genome curve generated by plotting total number of gene families in the pan and core genome for all isolates. **(B)** Polar diagram showing isolates having unique number of genes. 32 isolates out of 49 possess unique genes, represented in outer edge of the diagram. **(C)** Collective distribution of core, accessory and unique genes of all 49 isolates in COG and **(D)** KEGG analysis.

To study the genetic repertoire of the commensal *S. epidermidis* isolates', pan-genome analysis of 49 SEA and SEI genomes was performed using BPGA. The results of core pan curve showed that pan genome size increase with sequential addition of the new genome and reached a plateau state. The power-law regression model depicted “b” parameter value as 0.155528, suggesting an open pan genome, which may be closed soon. The analysis revealed that 49 compared *Staphylococcus epidermidis* strains possess 2178-2535 CDSs. Of these, 1,302 are core to all genomes in comparison (Figure 4A). However, nearly 700–1,000 CDSs proteins make up the accessory fraction, which include unique gene content to each genome among the 49 compared strains. Figure 4B depicts polar plot of pan-genome study with predicted number of core and unique genes across the 49 strains. It shows that, 32/49 strains are having a strain specific unique gene. The predicted genes (core, accessory and unique) in all 49 strains were further classified based on COG and KEGG categories. Functional classification according to COG category showed that the core gene pool was found to be abundant in all cell metabolism classes, ‘energy production, conversion, and translation, ribosomal structure and biogenesis. However, the unique gene pool of all the strains showed significant abundance of subclasses that belonged to transcription and replication, recombination and repair followed by cell motility and defense mechanisms compared to core (Figure 4C). As per KEGG category analysis (Figure 4D), the genome of all the strains depicted higher fraction of core genes under “metabolism” class whereas the unique gene pool were found high in number for drug resistance, immune system, infectious diseases, terpenoids and polyketides apart from amino acid, carbohydrate, xenobiotics metabolism and replication and repair genes.

#### Comparative analysis of functional gene classes

**Figure 5:**
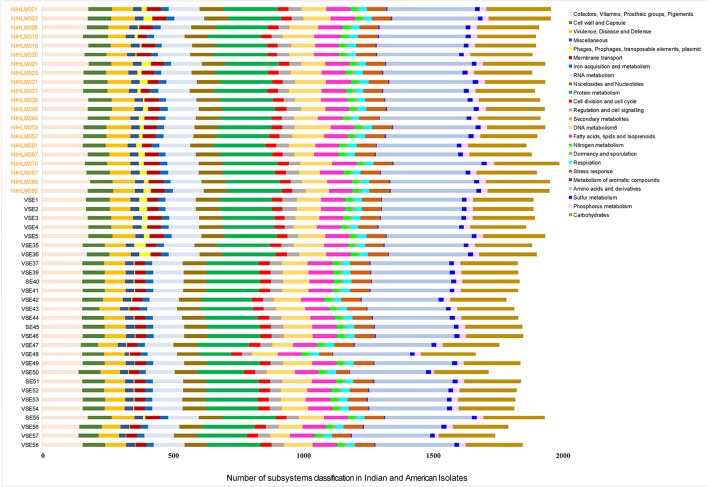
RAST Subsystem distribution analysis of SEI and SEA isolates of lineages, PG1 and PG2.

Functional comparisons of subsystems are similar in all the isolates, except variations in “phage, prophage, transposable elements, and plasmids” and “production of secondary metabolites” (Figure 5). Most of the SEI isolates of PG1 lineage showed fewer number of phages, prophages, transposable elements and plasmids as compared to SEA isolates of the same lineage while SEI isolates of PG2 lineage had higher number of phages, prophages, transposable elements and plasmids. However, phage presence could only affect the functionality of the genomes, if they are intact. Hence, an *in silico* analysis was carried out to figure out the exact intact phages in these genomes (Table S4). SEI isolates of lineage PG1, corroborates with the previous findings demonstrating absence of phages, except VSE35 and VSE36. Since, functional comparisons also revealed variations in secondary metabolite production, genomes were analyzed for the same. Presence of a lantibiotic, having similarity to “Epilancin 15x” and a sactipeptide, subtilosin A was detected in SEI isolates of PG1 lineage (Figure 3A, Table S5).

#### Mining atypical regions by genome wide comparisons

**Figure 6:**
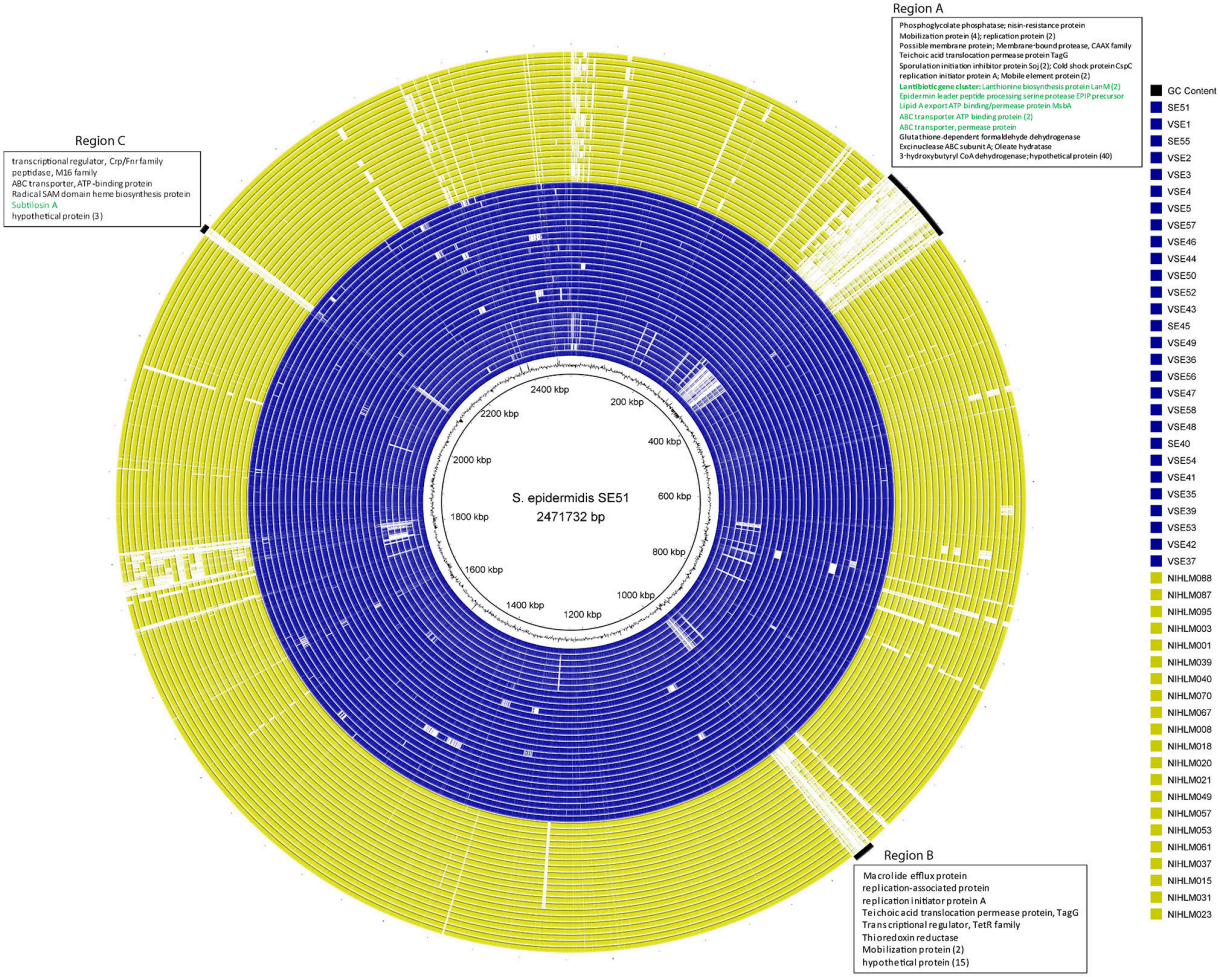
Blast based whole genome comparison of SEI and SEA isolates, taking Indian commensal, SE51 as reference. Blue color represents SEI isolates and yellow color represents SEA isolates. SEI isolates of PG1 lineage were seen to have three large dynamic regions with annotation indicated within boxes as region A, B, and C.

Whole genome comparisons were made to extract out atypical regions among genomes, taking different genomes as references. Three genomic regions (Region A, Region B, and Region C) were found to be unique to SEI isolates of PG1 lineage, against SE51 as reference (Figure 6).

**Figure 7:**
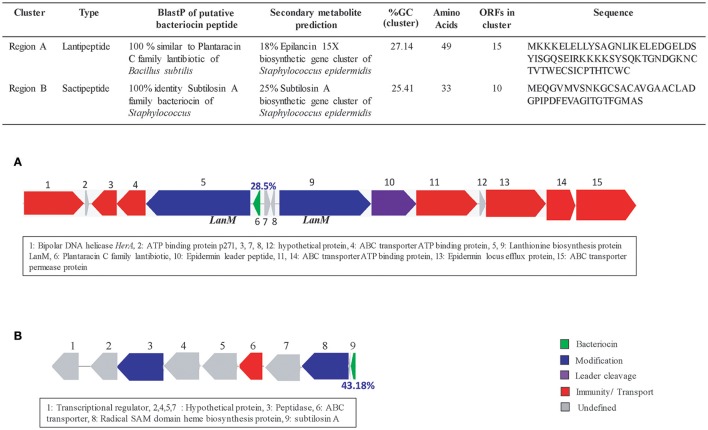
Bacteriocin details of Indian isolates from PG1 lineage. **(A)** Putative bacteriocin cluster from a “plantaracin C family” lantibiotic and **(B)** Putative bacteriocin, “subtilosin A” sactipeptide biosynthetic gene cluster.

Interestingly, region A, which was largest of all regions is about 65 kb with more than 70 predicted ORFs and 28.29% GC content, encoding for genes responsible for membrane proteins, mobile element proteins, antimicrobial peptide biosynthetic gene cluster, and large number of hypothetical proteins. Further annotation of this region revealed the presence of a lantibiotic peptide (49 aa), having 28.5% GC content and surrounded by LanM (lantibiotic biosynthesis enzyme) encoding genes (Table S6). BlastP of the peptide revealed its 100% similarity to “plantaracin C family lantibiotic” of *Bacillus subtilis*. Secondary metabolite prediction tools confirmed the presence of lantibiotic peptide and predicted a putative lantibiotic biosynthetic gene cluster having 18% homology to “Epilancin 15X biosynthetic gene cluster” (Velásquez et al., 2011). Putative lantibiotic biosynthetic gene cluster having the lantibiotic peptide and other associated genes were manually mapped (Figure 7A).

A comparatively smaller (19 kbp) Region B having 24 ORFs has a GC content of 30%. Most of the genes of this region are hypothetical proteins (15). However, one “macrolide efflux protein” gene was seen specific to the region (Table S7).

The smallest region (7.5 kb) having GC content of 25.42%, encodes for nine ORFs only, one of them which is for “subtilosin A” (Table S8). Performing BLASTN of the gene revealed its 100% identity to “serine protease” of *S. epidermidis* isolates and BlastP shows its identity to subtilosin A to *Staphylococcus*. Secondary metabolite prediction tools confirmed the presence of the gene by predicting a sactipeptide biosynthetic cluster having 25% homology to “subtilosinA biosynthetic gene cluster.” Putative sactipeptide biosynthetic gene cluster having the sactipeptide and its biosynthetic genes were manually mapped (Figure 7B). Complete details of all the genes of lantibiotic and sactipeptide clusters are present in Tables S9, S10 respectively.

Mapped putative lantibiotic and sactipeptide clusters were observed to be present only in SEI isolates of PG1 lineage. However, NIHLM040, a SEA was observed to have another lantipeptide of around 29 Kbp with high sequence identity of 90% to a lantipeptide isolated from “*Staphylococcus gallinarum*,” known as “Gallidermin” (Kellner et al., 1988). Clade specific comparative analysis revealed no significant cluster or gene, specific to any of the group, A or B. However, a small, 3.5 Kb region is specific to group A or lineage PG1 (Figure 3C) irrespective of geographic origin of the isolates. Annotation of this region revealed “formate dehydrogenase alpha subunit” i.e., *fdh*, followed by 2 hypothetical proteins (Table S11).

## Discussion

Microbial diversity is greatly affected by the environment and selection pressure. *S. epidermidis* isolates of Indian origin (SEI), used in this study may be diverse at genomic level but all are from a similar habitat. However, to depict the trend in their diversity *S. epidermidis* isolates from a completely diverse environment or niche are required. Therefore, taking advantage of an already existing dataset of 21 isolates of a study performed in North America i.e., at a different geographic location (Conlan et al., 2012), along with our own generated data. In this regard, we performed comparative genomic and phylogenomic analysis with multiple *S. epidermidis* North American isolates (SEA) collected from different body sites of healthy individuals.

Phylogenetic analysis depicts simultaneous diversification of the two lineages from a common ancestor. SEI and SEA isolates cluster themselves in two lineages, PG1 and PG2, independent of individual or site of isolation and geographic location. The ANI value difference of PG1 lineage isolates and PG2 lineage isolates varies considerably i.e., from 2 to 3%, suggesting that lineages are divergent from each other and their divergence is not recent. Presence of SEI isolates of PG1 lineage in a single group depicting their clonal nature, inspite of the fact that they were isolated from different individuals. However, SEI isolates of PG2 lineages are comparatively diverse. Based on ANI values, a minor third kind of population or transitional lineage highlighting SEI isolate VSE1, with three SEA isolates, depicts presence of three type of populations in commensal pool of *S. epidermidis*, at genomic level. Isolates collected from a single individual can cluster into both PG1 as well as PG2 lineages, suggesting that skin microbiota of a healthy individual generally can consists of 2-3 kinds of population irrespective of their geographical differences. However, there can also be individuals that harbor isolates from only one type of lineage or ST. Interestingly, individuals harboring isolates from only one lineage or ST are of Indian origin and not American origin. Clonal nature of SEI isolates of PG1 lineage is evident in their allelic profile, which was exactly same for all the isolates (Table S3). Presence of no exact matches for STs for these isolates in entire database of MLST, speculate presence of a single but novel ST in SEI isolates of PG1 lineage, which is might be unique to Indian population. In addition, presence of 19 STs in 21 SEA isolates signifies diversity of these isolates as compared to Indian ones (SEI).

### Revisiting commensal status of *S. epidermidis* isolates from healthy individuals

One of the biggest challenge of clinicians is to differentiate among pathogenic and commensal *S. epidermidis* due to lack of proper markers. Comparative analysis in some of the studies has highlighted that on the basis of their overall frequency in commensal and pathogenic isolates, some of the genes have the potential for being the discriminatory markers (Conlan et al., 2012). However, the scope of these genes is yet to be fully exploited and, hence, a clear and evident declaration of markers among the two kinds of *S. epidermidis* is lacking. In this study, we tried to evaluate the trend of these genes in two diverse kinds of population, i.e., Indian and western. The ecology, habitat, economical status and environment of these geographic populations is completely diverse. Trend of these genes in two different kinds of population will help in understanding the adaptation of these two different kinds of isolates and will throw light on the differentiating ability of these genes. Five potential marker genes: *fdh* (formate dehydrogenase), *arsD* (arsenic resistance cluster gene), IS*256* (insertion sequence), *icaA* (biofilm biosynthesis cluster *ica* gene), and *mecA* (methicillin resistant) were investigated for this purpose. Among these, *fdh* and *arsD* were found to be more common in commensal isolates while IS*256, icaA* and *mecA* were more frequent in clinical isolates (Conlan et al., 2012).

Presence of *fdh* gene in all isolates of PG1 lineage and their complete absence from that of PG2, reflects that distribution of this gene is lineage dependent, as reported by Conlan and co-workers, but lineage specific distribution is independent of geographic origin of the isolates, in our case SEA or SEI. Hence, it can be restated that “*fdh*” gene is associated with commensal strains of *S. epidermidis*. While its absence from pathogenic RP62A coincides with the hypothesis, its absence from non-biofilm forming ATCC12228 is counterintuitive. However, the commensal status of ATCC12228 strain is not clear as it harbors some of the virulence genes like “Delta haemolysin and beta haemolysin” and reported to have slight virulence in mouse and rats (Zhang et al., 2003). Another gene, *arsD* is also connected to commensalism. However, its absence from SEI isolates of lineage PG1 and inconsistent distribution among isolates of other lineages, contradicts its status as a demarking marker of commensal and pathogenic isolates. Since all isolates understudy are from healthy individuals, absence of IS*256* from majority of the isolates of all lineages is not surprising. Its presence in only two isolates could be a case of horizontal gene transfer, since both the isolates belong to the same individual. Antibiotic resistance is another important challenge in treating *S. epidermidis* infections. Methicillin resistant *S. aureus* (MRSA) is already known and studied extensively. Recent studies have reported many methicillin resistant *S. epidermidis* isolates (MRSE), further complicating the disease management. Pathogenic strain, RP62A is also known to harbor *mecA* gene that encodes for methicillin resistance. Presence of this gene in SEI isolates of PG2 lineage may be due to high antibiotic load in India (Laxminarayan and Chaudhury, 2016). Hence, presence of *mecA* gene in *S. epidermidis* in healthy individuals from India is a matter of concern. 3 SEA isolates of PG1 lineage, carrying the *mecA* gene, belongs to the same individual from which SEA isolate, NIHLM047 was isolated and similarly, all 4 SEI isolates from PG2 lineage carrying the *mecA* gene belongs to a single individual suggesting presence of this gene due to individual specific antibiotic resistance rather than overall load. One important factor required for pathogenicity is the ability to form biofilms. The *ica* cluster is found responsible for biofilm formation in pathogenic reference strain, RP62A (Li et al., 2005). The *ica* cluster (Heilmann et al., 1996), is followed by a tRNA in RP62A genome and has a low GC content (29.80%), suggesting that the cluster might have been acquired by the strain through horizontal gene transfer events. However, the whole cluster is fully conserved in all seven isolates of PG2 lineage, along with their locus. This implies that gain of the cluster is prior diversification of the two lineages. Occurrence of non-biofilm forming strain, ATCC12228 with other *S. epidermidis* isolates in lineage PG2 is not surprising as all are commensal bacteria. However, presence of pathogenic strain, RP62A in the same clade is unexpected, hinting at minor differences in commensal *S. epidermidis* isolates and RP62A at genomic level.

### Secondary metabolites and commensalism

Commensal *S. epidermidis* produces antimicrobials to inhibit the progression of pathogenic species, like *S. aureus* (Otto et al., 1999; Heikkilä and Saris, 2003; Iwase et al., 2010) and secondary metabolites for skin homeostasis (Lai et al., 2009; Belkaid and Segre, 2014; Wang et al., 2014) and stress tolerance (Gallo and Nakatsuji, 2011; Naik et al., 2012).

In fact, production of secondary metabolites is often related as a defined task of “being commensal” (Brestoff and Artis, 2013; Sharon et al., 2014). Presence of virulent or commensal genes focuses on the existences of two diverse phylogenetic lineages in commensal pool of *S. epidermidis*. However, presence of gene clusters for two antimicrobial peptides, a putative lantibiotic and sactipeptide, unique to SEI isolates on PG1 lineage, not only suggests commensal status of these isolates but also highlights diversity among SEA and SEI isolates belonging to same phylogenetic lineage but isolated from geographical locations. It is possible that the clusters have been acquired by horizontal gene transfer in recent past but after separation of these populations. One of the putative lantipeptide cluster showed 18% similarity to already identified lantipeptide from a clinical isolate of *S. epidermidis*, “Epilancin 15x,” which might have required the lantibiotic to survival and success. Presence of similar lantibiotic in commensal isolates of Indian origin, might play a role in ecological fitness of the isolates and their adaptation to stressed conditions like, humidity, high temperature etc. of the region. Existence of multiple secondary metabolite biosynthetic gene clusters is also supported by the fact that SEI isolates of PG1 lineage are clonal, which might maintains their clonality in order to conserve these metabolite gene clusters. Diverse SEA isolates might have lost these clusters due to frequent genome dynamics. In this context, it is surprising to note that a gene encoding macrolide-efflux protein is present only in SEI isolates from PG1 lineage. Further studies will allow us to understand selection pressure that has led to presence an antimicrobial resistance gene along with two antimicrobial peptide biosynthetic gene clusters in SEI isolates of PG1 lineage.

Presence of commensal specific marker, *fdh* in lineage PG1 and absence of virulent factors, *icaA* and IS*256* from all isolates from PG1, suggests lineage PG1 is comprised of “true commensals.” Non-biofilm forming strain, ATCC12228, present in PG2, is also devoid of commensal associated *fdh* gene. Though ATCC12228 is a non-biofilm producing strain and was isolated from a healthy individual, it does have many virulence factors (Zhang et al., 2003) and seems to have compromised commensal status. Presence of pathogenic RP62A strain in PG2 lineage, frequency of virulence factors and absence of commensal associated gene, “*fdh”* from transitional and PG2 lineage, suggests that these isolates are of varying pathogenicity, might have potential to turn into a pathogenic strain. In addition, where the study reasserts “*fdh”* as a putative marker to recognize a true commensal among pool of numerous and geographically diverse skin isolates, it restricts the use of “*arsD”* gene for the same purpose. In fact, absence of “*fdh”* gene, with presence of any one virulence gene can be considered sufficient to redefine that isolate as a “potential pathogenic strain.”

### Genome dynamics role in adaptation of *S. epidermidis*

Phage attack is one of the most important factor bringing out diversity in genomes and contributing to genomic evolution (Wei et al., 2008; Rodriguez-Valera et al., 2009; Stern and Sorek, 2011). Phage, prophage, transposable elements and plasmids not only bring genomic diversity but they are also the important source of antibiotic resistance genes and other virulence factors. Limited intensity of these in SEI isolates and their variation in the two lineages indicate mobilomes acquisition in *S. epidermidis* isolates, which is lineage as well as geography dependent. Isolates in PG2 and transitional lineage are commensals and presence of virulence factors might be an outcome of phage acquisitions.

Pangenome analysis revealing open pangenome suggest ongoing variation and selection in *S. epidermidis* strains. These results were in agreement with the previous pan genomic study on *S. epidermidis* (Conlan et al., 2012), which also showed the role of the above stated gene classes as evolutionary force that shape *S. epidermidis* genomes (Conlan et al., 2012). Further, core and unique genes were analyzed for KEGG pathway, which showed that high fraction of core belongs to metabolic pathways that are required for the normal functioning of the cell (Conlan et al., 2012). Further, unique gene pool were associated with antibiotic resistance, diseases, secondary metabolites apart from important metabolic pathways and, replication and repair processes. Hence, the genomic evidence explains the dual role of commensal *S. epidermidis* in human skin protection and as well as its conversion as a skin pathogen in disease. These findings provide new perspectives on the intra-strain diversity from phylogenomics point of view of important skin commensals in the human health and disease management. Unique gene analysis could not reveal any significant or identifiable region, gene or cluster unique to a special population but can be in a large sub-population, as seen in SEI of PG1 lineage. This suggests that may be evolution of both population is geographically independent and is rather undergoing parallel evolution, diversifying as two lineages.

Phylogenomic study comparison has revealed two diverse lineages of *S. epidermidis* in healthy individuals, depicting distinct pattern in geographically diverse *S. epidermidis* isolates with respect to candidate and novel genes. These genes may have a possible role in virulence, commensalism and adaptation to skin. In addition, a putative antimicrobial gene cluster was seen present in one type of population and strikingly absent in another, signifying the importance of systematic ongoing evolution of this organism having an important role in human health and disease. Though formation of two major lineages seems independent of individual and body site, isolates VSE2-VSE5, present in PG2 lineage are from a single individual F3 and are isolates from opisthenar part of the hand. None of the isolates from this site was observed to be present in PG1 lineage and all of them harbored one or more pathogenic markers. This implies precise yet noteworthy role of body site in pathogenicity of *S. epidermidis*. Isolates of this site might have been undergoing comparatively large selection pressure as compared to other body sites, as they are far more exposed in different adverse environmental conditions. Interestingly, the study highlighted two geographically different populations that are following similar evolution trend, diversified into different lineages, one of which consists of true commensals (PG1) and other consists of potential pathogenic strains (PG2). Potential marker genes trend in both lineages and conserved locus of *ica* gene cluster, highlights that both PG1 and PG2 lineage isolates are evolved from a single ancestor having all these marker genes. True commensals isolates lost the pathogenic genes and retained the commensal marker gene, *fdh*, while PG2 isolates, lost the commensal markers and retained the pathogenic ones. Since, similar evolution trend is observed in both Indian and American isolates; it suggests an ongoing parallel evolution in *S. epidermidis* (Bailey et al., 2017).

Occurrence of some pathogenic strains in the otherwise commensal species, *S. epidermidis*, pose it as an accidental pathogen, which might have gained some of the virulence factors by horizontal gene transfer. However, presence of multiple lineages in *S. epidermidis* isolates collected from healthy individuals, independent of geography, individual or site of isolation, forces us to revisit the current hypothesis. The study mentions two major lineages, one of which can be regarded as “true commensal”; since it is devoid of any pathogenic, genes and another one contain strains which might hold the potential to be pathogenic. This suggests that occurrence of some pathogenic strains in this species might not be accidental and rather, is a result of ongoing negative frequency selection, shaping a diverse, disease-causing lineage among the commensal pool of *S. epidermidis*. Similar kind of results has been reported in many recent studies (Takeuchi et al., 2015; Corander et al., 2017). However, a detailed analysis including a large dataset, including global isolates and multiple factors (geography, site of isolation) is required to actually validate this hypothesis.

## Conclusion

Our phylogeographic study has revealed that the *S. epidermidis* population harbored by healthy individuals consist of at least two phylogenomically divergent lineages, suggesting parallel evolution of a disease causing or opportunistic lineage. This can be seen in the distinct pattern in distribution of candidate gene(s) for pathogenicity and commensalism. This is reflected in the fact the SEA isolates understudy are more genomically dynamic and harbored pathogenicity markers in higher frequency On the other hand, isolates of Indian origin are less genomically dynamic, harbored less pathogenicity marker gene and have multiple unique antimicrobial peptide biosynthetic gene clusters. Further studies to understand the selection pressure for such systematic variation, having implication in *S. epidermidis* lifestyle, as an evolving pathogen or beneficial skin probiotic is required, to explore its potential in disease management.

## Author contributions

SS and VC carried out isolation of strains. SS and VC are involved in genome sequencing, submission and analysis. SS, VC and SK were involved in *insilico* analysis of the data. VC, SS, and PP participated in conception, design of the study, interpretation of data and drafting of the manuscript. PP applied for funding. All authors reviewed and approved the manuscript.

### Conflict of interest statement

The authors declare that the research was conducted in the absence of any commercial or financial relationships that could be construed as a potential conflict of interest.
